# Early Life Stress Enhancement of Limbic Epileptogenesis in Adult Rats: Mechanistic Insights

**DOI:** 10.1371/journal.pone.0024033

**Published:** 2011-09-21

**Authors:** Gaurav Kumar, Nigel C. Jones, Margaret J. Morris, Sandra Rees, Terence J. O'Brien, Michael R. Salzberg

**Affiliations:** 1 Department of Medicine, Royal Melbourne Hospital, University of Melbourne, Parkville, Australia; 2 Department of Pharmacology, School of Medical Sciences, University of New South Wales, Sydney, Australia; 3 Department of Anatomy and Cell Biology, University of Melbourne, Parkville, Australia; 4 Department of Neurology, Royal Melbourne Hospital, University of Melbourne, Parkville, Australia; 5 Department of Surgery, Royal Melbourne Hospital, University of Melbourne, Parkville, Australia; 6 St. Vincent's Mental Health Service, St. Vincent's Hospital, Fitzroy, Australia; 7 Department of Psychiatry, St. Vincent's Hospital, University of Melbourne, Fitzroy, Australia; McGill University, Canada

## Abstract

**Background:**

Exposure to early postnatal stress is known to hasten the progression of kindling epileptogenesis in adult rats. Despite the significance of this for understanding mesial temporal lobe epilepsy (MTLE) and its associated psychopathology, research findings regarding underlying mechanisms are sparse. Of several possibilities, one important candidate mechanism is early life ‘programming’ of the hypothalamic-pituitary-adrenal (HPA) axis by postnatal stress. Elevated corticosterone (CORT) in turn has consequences for neurogenesis and cell death relevant to epileptogenesis. Here we tested the hypotheses that MS would augment seizure-related corticosterone (CORT) release and enhance neuroplastic changes in the hippocampus.

**Methodology/Principal Findings:**

Eight-week old Wistar rats, previously exposed on postnatal days 2–14 to either maternal separation stress (MS) or control brief early handling (EH), underwent rapid amygdala kindling. We measured seizure-induced serum CORT levels and post-kindling neurogenesis (using BrdU). Three weeks post-kindling, rats were euthanized for histology of the hippocampal CA3c region (pyramidal cell counts) and dentate gyrus (DG) (to count BrdU-labelled cells and measure mossy fibre sprouting). As in our previous studies, rats exposed to MS had accelerated kindling rates in adulthood. Female MS rats had heightened CORT responses during and after kindling (p<0.05), with a similar trend in males. In both sexes total CA3c pyramidal cell numbers were reduced in MS vs. EH rats post-kindling (p = 0.002). Dentate granule cell neurogenesis in female rats was significantly increased post-kindling in MS vs. EH rats.

**Conclusions/Significance:**

These data demonstrate that early life stress results in enduring enhancement of HPA axis responses to limbic seizures, with increased hippocampal CA3c cell loss and augmented neurogenesis, in a sex-dependent pattern. This implicates important candidate mechanisms through which early life stress may promote vulnerability to limbic epileptogenesis in rats as well as to human MTLE and its associated psychiatric disorders.

## Introduction

Mesial temporal lobe epilepsy (MTLE), the most common form of focal treatment-refractory epilepsy in adults [1], arises from limbic structures highly sensitive to the effects of stress, notably the hippocampus [2]. Thus stress may be relevant to the causation both of MTLE and of its psychiatric comorbidities, such as depression [3]. Indeed, there is good experimental evidence that stressors can affect several stages in the multi-step pathogenesis of MTLE, which is thought to commence in early life [4], [5]. For stressors in adult life, studies are consistent in reporting that stress or stress mediators enhance epileptogenesis in experimental models [6]. For example, administering exogenous corticosterone (CORT) aggravates kindling epileptogenesis [7], [8], [9], an effect reversed using antagonists of glucocorticoid and mineralocorticoid receptors [8]; and kindling is slowed in adrenalectomised or hypophysectomised rats [10], [11], [12], [13]. Recently it has become increasingly clear that stress in early life can result in enduring vulnerability to epileptogenesis in adult animals [14], [15], [16], however, experimental data about mechanisms underlying such effects remain sparse.

For stress both in adult and early life there are several candidate mechanisms, as stress affects a range of neurobiological structures and functions relevant to epileptogenesis [2], [4], [17], [18], [19], including dendritic structure in the hippocampus and amygdala [2], [19], hippocampal neurogenesis [20], electrophysiological function [21], [22], and neurochemical systems [17]. For stress in adult life, valuable progress has been made in examining these mechanisms [6], but for early life stress very little experimental data are available about intervening mechanisms in epilepsy models [17].

Previously we demonstrated that maternal separation (MS) stress at postnatal days 2–14 resulted in accelerated electrical amygdala kindling in adult rats [15], [23]. MS was chosen for this research as it is a well-established, extensively studied form of moderate-to-severe early life stress, with enduring effects on limbic neurodevelopment and the HPA axis [17], [24]. PN2-14 in the rodent is a time when structures important to limbic epilepsy, namely the hippocampus and amygdala, are developing [25], [26], [27]. Here we aimed to examine the involvement of two candidate intermediary processes through which early life stress might result in enduring increased vulnerability to limbic epileptogenesis. Specifically, these were seizure-associated CORT release and key aspects of hippocampal neuroplasticity, namely dentate gyrus (DG) neurogenesis, synaptogenesis and pyramidal cell loss. Based on studies showing ‘programming’ effects of early life stress on HPA axis function [28], [29], we hypothesized that, in maternally separated rats, seizure-associated CORT release would be elevated. Additionally, we hypothesized that MS stress would be associated with enhanced kindling-associated dentate gyrus neurogenesis and with increased pyramidal cell loss in the CA3c region of hippocampus, two regions believed to be pivotally implicated in MTLE pathogenesis. These predictions were borne out by the experimental results, but in a sexually dimorphic manner, being consistently found in female animals to a statistically significant degree with males also showing a significant reduction in CA3c cell counts, but non-significant trends for the other main outcome measures.

## Materials and Methods

### Experimental Animals

Male and female Wistar rats were bred and housed in the Department of Zoology Animal House Facility, maintained at 24°C on a 12 hr light/dark cycle (lights on 0600hr), with rat chow and water available *ad libitum*. At age 6 weeks, all were transferred to the Department of Medicine, Royal Melbourne Hospital Biological Research Facility for all experiments. The study was approved by The University of Melbourne Animal Ethics Committees. Following up our previous work, [15], we repeated our kindling studies using new cohorts and in addition measured: CORT responses during kindling, BrdU-defined neurogenesis, and pyramidal cell counts in the hippocampal CA3c region. We used rats from 7 litters, all of 8–12 pups (65 pups in total). The average litter size did not differ between MS and ‘early handling’ (EH) groups.

### Early Life Interventions and Electrode Implantation

The methods for EH and MS have been described previously [15]. Briefly, litters were randomly assigned to the two protocols: daily, from P2-14 inclusive, rats were handled and separated from dams for either 15 minutes (800 to 815 hr; EH) or for 180 minutes (800 to 1100 hr; MS), then returned to the dam's cage. At 7 weeks of age, rats were anesthetized with a mixture of xylazine (Troy Laboratories, 10 mg/kg) and ketamine (Parnell Laboratories, 75 mg/kg) in 0.9% saline (ip). The skull was then exposed via a single midline incision and five holes drilled to accommodate three gold ‘male’ connector electrodes (Farnell In One) previously soldered onto nickel alloy jewellers' screws, and one jewellers' screw to serve as ground reference electrodes and anchoring respectively. A bipolar electrode (Plastics One) was then inserted into the left amygdala using coordinates (AP: −3.0; ML: +5.0 relative to bregma; DV: −6.5 relative to dura) [30]. All electrodes were secured with dental cement.

### Afterdischarge Threshold Measurement and Rapid Amygdala Kindling

After one week recovery from surgery, rats were tested for an afterdischarge prior to rapid amygdala kindling, as described previously [8]. Briefly, an electrical stimulus of 1-sec duration was delivered to the bipolar electrode (Accupulser Pulse Generator/Stimulator (A310), WPI, Sarasota, FL, U.S.A.), initially at a current of 20 µA, incrementing by 20 µA until an afterdischarge of at least 6 s duration was observed on the EEG (Compumedics, Melbourne, Australia). If a stimulus amplitude of 400 µA did not evoke an afterdischarge, we assumed the electrode was incorrectly placed. All rats that had an afterdischarge were randomly divided equally into either sham or kindled subgroups of the two interventions. Subjects without an afterdischarge were utilized as additional shams (n = 6) for CORT experiments (see below).

The rapid amygdala kindling protocol employed stimulations (fixed 10 s, 60 Hz trains of 1 ms biphasic square wave pulses at 400 µA) administered via the bipolar electrode up to 24 times per day, with 15–20 minutes inter-stimulation intervals and 1–2 days between stimulation days until the fully kindled state was reached (i.e. 5 class V seizures [31]). Sham kindling involved identical handling without electrical stimulation.

### Blood Sampling and CORT Measurement

Rats were gently restrained and tail vein serial blood samples (0.1 ml) taken using heparinised syringes. Samples were acquired before kindling (0900–1000 hr), and shortly after (<5 min) the 12^th^ and 24^th^ electrical stimulation, or sham stimulation. Two weeks following full kindling, a further stimulus was administered to elicit a sixth class V seizure (which occurred in every case), followed by another blood sample. Blood was centrifuged at 4°C, and serum isolated and stored at −20°C until assayed. The highly specific radioimmunoassay was performed to manufacturer's instructions (MP Biomedical, OH, US). All samples were processed in duplicate and inter- and intra-assay variability was below 10%.

### BrdU, NeuN and Synaptophysin Immunohistochemistry

To label new born cells in the DG, rats were administered 7 daily injections of BrdU (5-bromo-2-deoxyuridine, 50 mg/kg ip dissolved in saline) (Sigma St Louis, MO, USA) beginning on the final day of kindling after the 5^th^ Class V seizure. Two weeks after the last BrdU injection, rats were transcardially perfused with 4% paraformaldehyde, brains removed and frozen in cryoprotectant. Serial coronal sections (20 µm) encompassing the hippocampus were cut on a cryostat, slide-mounted and stored at −80°C until further use.

The staining protocol was adapted from previous studies [32], [33], [34], [35]. Briefly, sections were washed with 0.1 M TBS (Tris buffer saline, pH 7.4) and microwave antigen retrieval performed in a 0.1 M citrate buffer at pH 6.0. Sections were rinsed in 1% hydrogen peroxide in 50% methanol, and, for BrdU staining only, incubated in 2 N HCl (37°C) to denature DNA, then neutralized with 0.1 M sodium borate (pH 8.5). Sections were incubated overnight at 4°C with primary antisera: monoclonal rat anti-BrdU (Accurate Chemical, Westbury, NY; 1∶400), monoclonal mouse anti-NeuN (Chemicon, Temecula, CA; 1∶200), or monoclonal mouse anti-synaptophysin (Sigma, USA; 1∶200), all diluted in 0.3% Triton X100 in 0.1 M TBS. This was followed by 2 hours incubation in appropriate biotinylated secondary antibodies: goat anti-rat (Vector Laboratories, Burlingame, CA, USA; 1∶400), goat anti-mouse (Vector Laboratories, Burlingame, CA, USA; 1∶200), all diluted in 4% NGS, followed by incubation in avidin-biotin-peroxidase (Vectastain ABC-Kit, Vector Laboratories), and then diaminobenzidine (MP Biomedicals, Solon, OH, USA). These sections were used for localising BrdU+ve cells, counting of CA3c neurons, and assessing synaptophysin expression, as appropriate. Digital images of synaptophysin-stained sections were taken at 100× magnification and relative optical density (ROD) was measured in the CA3c region using ImageJ software (NIH) [36].

### BrdU, NeuN, and GFAP Fluorescent Immunohistochemistry

To determine the phenotype of BrdU+ve cells, sections from MS and EH female rats were used. Four coronal sections randomly selected between bregma −3.60 and −4.80 [30] were processed for fluorescent immunohistochemistry labelling of BrdU (newly generated cells), NeuN (neurons), and GFAP (glia). Sections were incubated overnight (18–20 h) at 4°C with all three primary antibodies: monoclonal rat anti-BrdU (Accurate Chemical, Westbury, NY; 1∶400); monoclonal mouse anti-NeuN (Chemicon, Temecula, CA; 1∶200); and polyclonal rabbit anti-GFAP (Dako, USA; 1∶200). The following day, all tissue sections were washed three times in TBS with 0.1% Tween 20 (TBST) and incubated for 2 h in three fluorophore-labelled secondary antibodies: Alexa 405 goat anti-rabbit (1∶200); Alexa 594 goat anti-rat (1∶400); and Alexa Fluor 488 goat anti-mouse (cross-adsorbed, 1∶200) (Invitrogen, USA) diluted in 4% NGS in TBS with 0.3% Triton X-100. Sections were rinsed in TBST, and cover slipped with mounting media (DAKO, Denmark). Controls for the fluorescent immunohistochemical procedures consisted of omitting the primary antibody from the protocol. No specific BrdU, NeuN, or GFAP immunoreactivity was observed under any of these control conditions.

### Timm's Staining of Mossy Fibres

Timm's staining was performed as previously described [37]. Briefly, for each animal, 4 coronal sections were randomly selected between bregma −3.60 and −4.80, exposed to hydrogen sulphide gas in a sealed chamber for 3 hours and developed in Timm's solution composed of 50% solution of acacia gum, citrate buffer, hydroquinone solution and AgNO3. Slides were developed at 37°C for 120 min in the dark, then dehydrated and cover slipped with mounting media. The inner molecular layer (IML) of the DG, and stratum oriens at CA3 were outlined with ImageJ software (NIH), and relative optical density of regions of interest were calculated as the optical density of the stratum oriens or IML divided by optical density of the stratum radiatum (background).

### Stereological Quantifications of Pyramidal Cell Numbers in the Hippocampal CA3c Region

Neuronal numbers were estimated in the ipsilateral CA3c region of the hippocampus using an optical fractionator [38], with CA3c defined as the terminal portion of CA3 inside the two blades of the DG. Counts were performed on every twentieth section collected from a series of NeuN-labelled sections cut between bregma −2.80 mm to −6.80 mm [30]. Stereoinvestigator software (MicroBrightField, VT, USA) interfacing with an Olympus BX51 microscope (PA, USA), position encoders (MAC 5000) and a digital video camera was used to estimate neuronal numbers. The regions of interest were first delineated using 20× objective lens at 100× magnification. Neurons were then viewed under oil immersion (100× oil objective lens; NA1.4). The dimensions of the optical fractionator counting frame, i.e. optical dissector, were 60 µm×60 µm×8 µm with a grid size of dimension 130 µm×130 µm. These parameters were chosen to ensure a suitable number of cells were counted (at least 200) to minimize the effect of inter-section variability and reduce error. Counting was performed by a single investigator blinded to treatment group. The precision of estimates of neurons in a set of sections for each animal was expressed using coefficients of error (CE). The stereological sampling scheme was considered adequate when the CE was less than 0.10 [39]. We also used thionin-stained brain sections of kindled rats to verify correct electrode placement in the left amygdala complex [30]. The placement in one animal (from the EH group) was found to be incorrect, and the animal was removed from all analyses.

### BrdU Cell Counts

Every twentieth brain section was used to count the number of BrdU+ve cells in the granule cell layer (GCL) and the subgranular zone (SGZ), defined as a two-cell body thick layer between the GCL and hilus. Only medium and large stained nuclei located in the defined region were counted. Rod shaped endothelial-like and small BrdU+ve nuclei, suggestive of glial cells and/or precursors (approximately<5 µm in diameter) were excluded from analysis, as previously described [35].

To estimate the total number of BrdU+ve cells in the ipsilateral hippocampus, we used a method modified from that described previously [40] using Stereoinvestigator software (MicroBrightField, VT, USA). On a live digitalised microscope image, GCL and SGZ regions were delineated using a 20× objective lens and all BrdU+ve cells within these regions were counted at a magnification of 100×. We then multiplied the number of BrdU+ve cells by 20 to estimate the total number of cells in each ipsilateral DG.

### Confocal Microscopy

Immunofluorescence studies were analysed using Olympus Fluoview software interfaced with an FV1000 motorized inverted laser scanning confocal microscope. Images from the three laser channels were combined using confocal Fluoview software. Several images of the DG region were taken to include the entire DG region of each section into the analysis. Images were examined for co-expression of (1) BrdU and NeuN (a neuronal phenotype), (2) BrdU and GFAP (a GFAP+ve glial phenotype) and (3) BrdU only (neither NeuN- nor GFAP-co-labelled). From these data, percentages were determined for the number of BrdU-NeuN+ve neurons, BrdU-GFAP+ve cells, and BrdU+ve only.

### Statistical Analyses

All quantitative histological analyses were performed on coded slides with the investigator blind to treatment group. For statistical testing we utilised Statistica® software (Tulsa, OK). Afterdischarge thresholds were analysed using one-tailed Student's t-test. The numbers of stimulations required to reach each of the 5 stages of kindling were compared between the groups using one-way ANOVA with repeated measures followed by planned comparisons. CA3c neuronal numbers and BrdU+ve cell numbers were analysed with two-way ANOVA, with early life interventions and kindling as independent variables and, if a significant effect was found, this was followed by planned comparisons to compare for differences between specific groups. Proportions of BrdU+ve cells co-localizing with NeuN in kindled rats were analysed using the Chi-square test. CORT responses were compared using ANOVA with repeated measures (time), and, if appropriate, a planned comparison at each time point. Data were analysed separately for each sex. Subsequently, sex differences were analysed using a three-way ANOVA with sex, kindling and early life interventions as the independent variables. The data are presented as mean ± SEM unless otherwise stated.

## Results

### Rats Subjected to MS Have Reduced Afterdischarge Thresholds and Enhanced Rates of Amygdala Kindling in Adulthood

**Figure 1:**
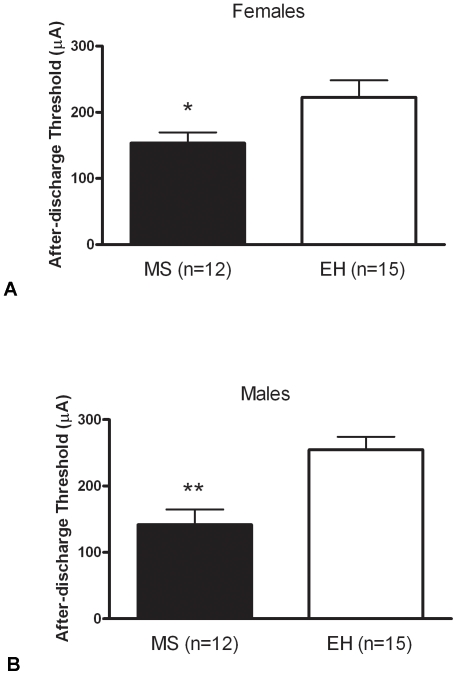
Maternal separation reduces after-discharge threshold in adult rats. Female (A) and male (B) rats exposed to maternal separation (MS – solid bars) display increases in limbic excitability compared with rats exposed to early handling (EH – open bars), as evidenced by significantly reduced after-discharge threshold (*p<0.05). Data represent mean ± SEM, numbers in each group indicated in parentheses.

The afterdischarge threshold was significantly lower in female MS (n = 12) rats compared to EH (n = 15) (t = 2.142, p = 0.042; Fig. 1A), and a similar effect was observed for males: MS (n = 12) vs. EH (n = 15) (t = 3.783, p<0.001; Fig. 1B). Combined analysis of males and females revealed no sex effect (F = 0.210, p = 0.646).

**Figure 2:**
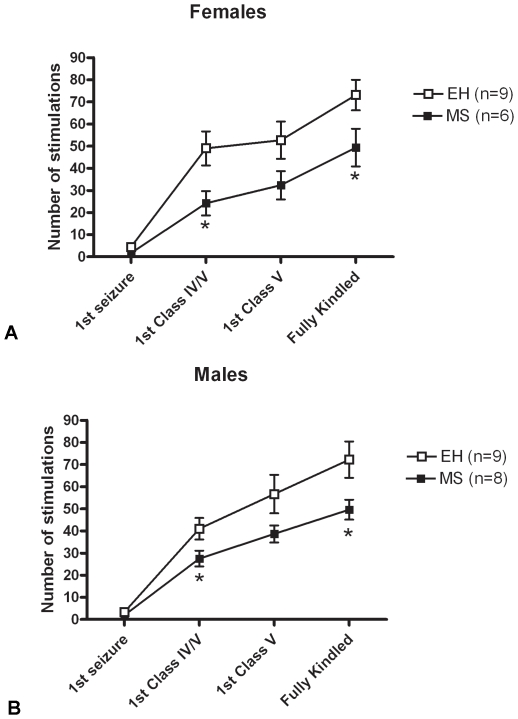
Maternal separation accelerates amygdala kindling rates in adult rats. Female (A) and male (B) rats exposed to maternal separation (MS – closed boxes) display enhanced vulnerability to the progression of limbic epilepsy compared with rats exposed to early handling (EH - open boxes), as evidenced by significantly reduced number of electrical stimulations required to reach the different stages of kindling (*p<0.05). Data represent mean ± SEM, numbers in each group indicated in parentheses.

Consistent with our previous report, female MS rats (n = 6) displayed significantly enhanced kindling rates compared to female EH rats (n = 9) (F = 4.767, p = 0.048; Fig. 2A), and a similar effect in males: male MS rats (n = 6) kindled faster than male EH rats (n = 9) (F = 4.879, p = 0.043; Fig. 2B). An ANOVA on the combined data revealed a highly significant overall effect of early life intervention (F = 9.714, p = 0.004), but no overall sex effect (F = 0.034, p = 0.856).

### Rats Subjected to MS Have Enhanced CORT Responses to Kindled Seizures

**Figure 3:**
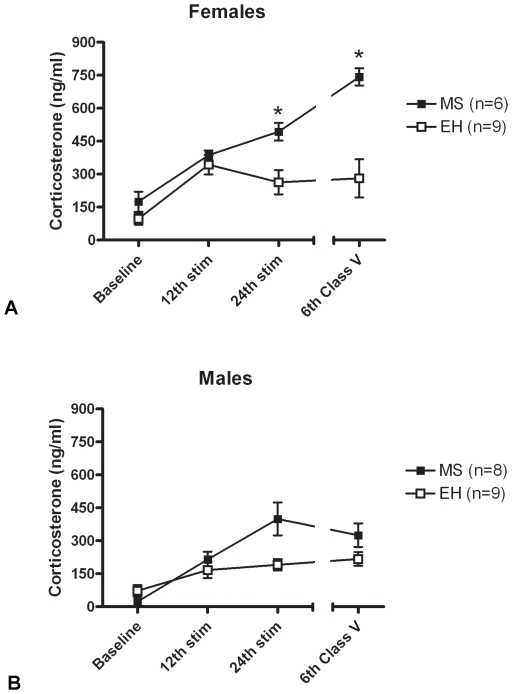
Maternal separation enhances seizure-induced corticosterone release in adult rats. (A) Female rats exposed to maternal separation (MS – closed boxes) display HPA axis hyper-responsivity to seizures compared with rats exposed to early handling (EH – open boxes), as evidenced by significantly enhanced corticosterone release following seizures (*p<0.05). (B) In males, a similar trend was observed, but this did not reach statistical significance. Data represent mean ± SEM, numbers in each group indicated in parentheses.

Female MS rats (n = 6) had higher serum CORT levels in response to seizures compared to EH rats (n = 9) (F = 23.81, p = 0.0006) throughout the kindling process, and this reached *post hoc* significance following the 24^th^ stimulation and the 6^th^ Class V seizure (p<0.05) (Fig. 3A). In male MS rats, serum CORT levels were higher compared to EH rats, but this failed to reach significance (F = 3.34, p = 0.09, Fig. 3B). Overall, in sham-kindled rats CORT levels in MS and EH groups of both sexes were not different from each other (females: F = 0.369, p = 0.547; males: F = 0.371, p = 0.546) (data not shown.).

### Rats Subjected to MS Have Reduced Neuronal Numbers in CA3c Subregion of Hippocampus Post-Kindling

**Figure 4:**
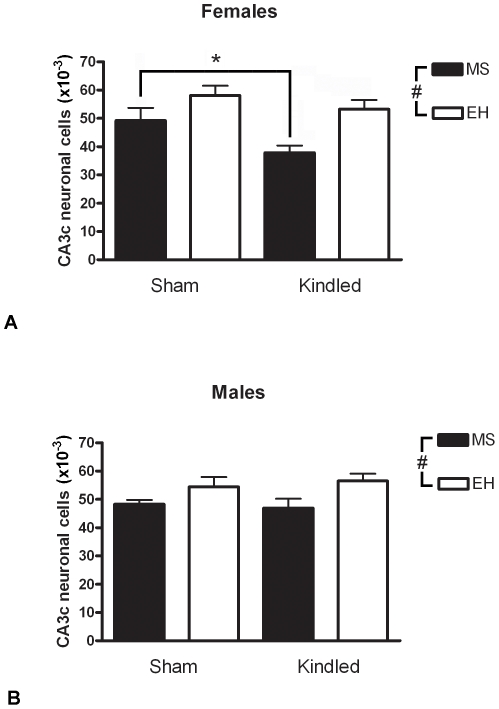
Maternal separation reduces hippocampal pyramidal cell counts. Female (A) and male (B) rats exposed to maternal separation (MS – closed bars) display significantly reduced CA3c pyramidal cell numbers compared with rats exposed to early handling (EH – open bars) (ANOVA; # p<0.05). Furthermore, in females (but not males), amygdala kindling results in reduced neuronal counts in this region, and this reached statistical significance only in rats exposed to maternal separation (* p<0.05). Data represent mean ± SEM. Numbers in each group: Female MS kindled: n = 6, sham kindled: n = 5; Female EH kindled: n = 9, sham kindled: n = 8; Male MS kindled: n = 8, sham kindled: n = 7; Male EH kindled: n = 9, sham kindled: n = 6.

Overall, pyramidal cell counts in CA3c were reduced in MS rats compared with EH rats in females (F = 11.63, p = 0.002; Fig. 4A) and males (F = 7.61, p = 0.01; Fig. 4B). Kindling was associated with significantly reduced cell numbers in MS-exposed female rats (F = 5.19, p = 0.031, *post hoc* planned comparison p<0.05), but not in EH-exposed female rats (p>0.05). In males kindling had no overall effect on CA3c cell counts (F = 0.02, p = 0.89). An ANOVA on the combined data revealed no overall effect of sex (F = 0.729, p = 0.40). The estimated coefficient of error (Schaffer CE) was below 0.10 in all cases.

### Rats Subjected to MS Have Increased Neurogenesis in Dentate Gyrus Subregion of Hippocampus Post-Kindling

**Figure 5:**
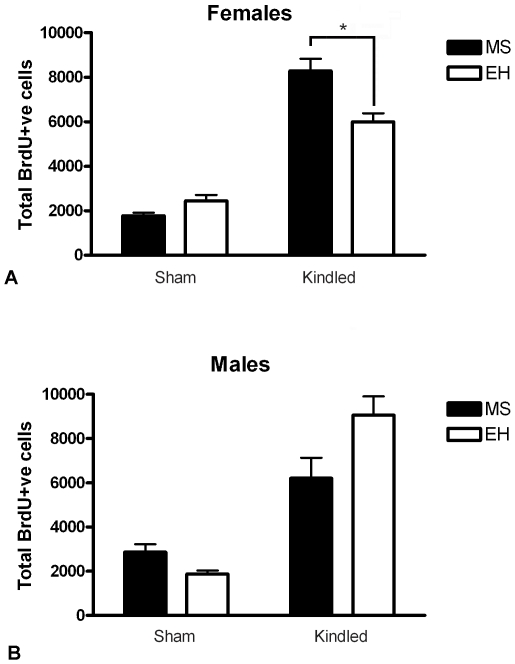
Maternal separation enhances neurogenesis in female, but not male, rats following amygdala kindling. (A) Female rats exposed to maternal separation (MS – closed bars) display significantly increased BrdU+ve cells in the dentate gyrus compared with rats exposed to early handling (EH – open bars), and *post hoc* analysis revealed this to be significant in amygdala-kindled female rats exposed to MS (* p<0.05). (B) In males, kindling also increased neurogenesis, but no significant differences were observed between MS- and EH-exposed rats. Data represent mean ± SEM.

Kindling induced a highly significant increase in neurogenesis in female (F = 173.7, p<0.0001; Fig. 5A) and male (F = 49.6, p<0.0001; Fig. 5B) rats compared to sham-kindled controls, as assessed by BrdU labelling. Female rats exposed to MS showed significantly greater levels of neurogenesis compared to EH (F = 11.27, p = 0.002; Fig. 5A), an effect observed only in the kindled group where MS-kindled rats had elevated neurogenesis compared with EH-kindled rats (*post hoc* planned comparison p<0.05). No differences in baseline neurogenesis (i.e., sham kindling) were observed in females (p>0.05). As with seizure-induced CORT responses and CA3c cell counts, this effect of MS was significant only in females, with no statistically significant difference in neurogenesis observed between male MS and EH rats (F = 1.570, p = 0.221; Fig. 5B). An ANOVA on the combined data revealed no overall effect of sex (F = 0.012, p = 0.911).

**Figure 6:**
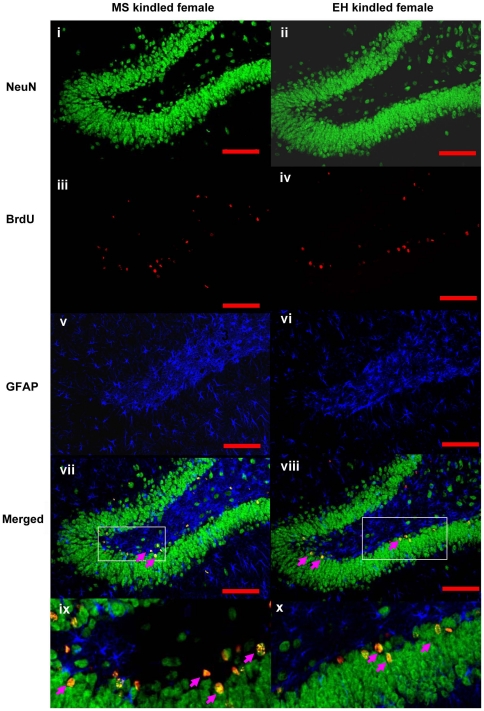
BrdU-labelled cells predominantly adopt a neuronal fate. Images show representative images from a MS-exposed female kindled rat brain (left panels) and an EH-exposed kindled female rat brain (right panels). Panels (i) and (ii) display NeuN+ve cells in green, indicative of mature neurons. Panels (iii) and (iv) display BrdU+ve cells in red, indicative of newly synthesised cells. Panels (v) and (vi) represent GFAP+ve glial cells in blue. Panels (vii) and (viii) represent the merged images of the three panels above, and panels (ix) and (x) are enlarged sections of the boxed areas in panels (vii) and (viii), respectively. Note the pink arrows indicating cells yellow cells in panels (vii) – (x), the product of double-labelling with NeuN (green) and BrdU (red), and indicative of newly generated neurons.

**Figure 7:**
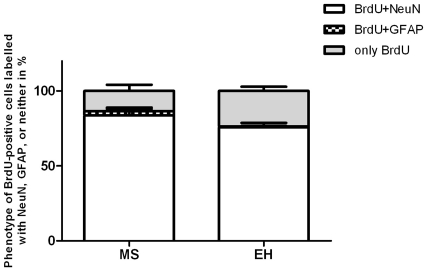
Phenotypes of BrdU positive cells labelled with NeuN, GFAP or neither in kindled MS and EH rats (p<0.05).

Confocal microscopy of the immunofluorescently stained cells revealed that most newborn cells (BrdU+ve) in both MS and EH groups co-localized with NeuN (**see** **Fig. 6**), a marker of mature neurons (83% in MS kindled vs 75% in EH kindled rats; **p<0.05**, **Fig. 7**). Only rarely did BrdU+ve cells co-localise with GFAP, a glial cell marker, consistent with previous reports [41].

### MS and Kindling Epileptogenesis Do Not Affect Measures of Synaptic and Axonal Reorganization in the Hippocampus

Mossy fibre sprouting (MFS) in the inner molecular layer of the DG was not significantly different between sham vs kindled rats (female: F = 1.119, p = 0.301; male: F = 1.015, p = 0.321), or between rats exposed to MS vs. EH (female: F = 0.311, p = 0.582; male: F = 0.048, p = 0.832) for either sex. There were also no differences observed in any comparison when assessing sprouting in the striatum oriens of CA3 (data not shown). Likewise, no differences were observed in synaptophysin immunostaining in CA3c between sham vs. kindled rats (female: F = 0.048, p = 0.828; male: F = 3.503, p = 0.078), or MS vs. EH-exposed rats (female: F = 2.151, p = 0.160; male: F = 0.461, p = 0.506).

## Discussion

Here we replicated our previous finding that adult rats of both sexes subjected to maternal separation stress exhibit enhanced kindling epileptogenesis, then extended this with novel observations relevant to underlying mechanisms: first, we found that accelerated epileptogenesis is accompanied, in female rats, by higher seizure-induced CORT levels, with a similar trend in males, and with increased hippocampal dentate gyrus neurogenesis; and, second, in both sexes we found decreased CA3c pyramidal cell numbers. Together with other structures, these hippocampal subregions - dentate gyrus and CA3c subfield - are considered pivotal to the pathogenesis of MTLE [4], [42]. These structures are intimately interconnected [43], [44] and, together with other hippocampal subfields, CA3c is vulnerable to amygdala kindling-induced cell loss [45]. In explaining why early life stress promotes vulnerability to limbic epilepsy, our data suggest important candidate mechanisms with which to guide future experimental research.

### Exposure to Early Life Stress Is Associated with Enduring Enhanced CORT Release following Kindled Seizures

Typically, early life stress ‘programming’ of the HPA axis results in enduring enhanced CORT responses to stressors [46], [47]. As predicted, we observed in MS-exposed rats enhancement of CORT response to kindled seizures, to a statistically significant extent in females and with a similar but non-significant trend in males. CORT release following seizures could be due to a physiological stress response and/or to seizure-related excitation of the paraventricular nucleus via amygdala [48]. Consistent with previous studies of MS [46], [47], we found no differences in baseline CORT levels between either sham-kindled MS and EH groups or in the pre-kindling assays in MS and EH groups.

A great deal of evidence already implicates CORT in the mechanism connecting stress in adulthood to epileptogenesis [49]: elevating CORT augments epileptogenesis and seizures in several animal models [7], [8], [9], [13], [50], [51], [52], [53], an effect reversed by antagonist of glucocorticoid and mineralocorticoid receptors [8]; whereas kindling is slowed in adrenalectomised or hypophysectomised rats [10], [11], [12], [13]. Thus, a direct effect of the enhanced CORT release post-seizures is a strong candidate explanation of the accelerated kindling epileptogenesis in MS exposed rats. How elevated CORT might exert this action remains an open question for future research. Elevated CORT has relevant effects on neuronal excitability [21], [49], on neurogenesis [20] and on risk of excitotoxic cell loss [54], [55]. In addition, elevated CORT results in elevation of corticotrophin releasing hormone (CRH) in amygdala and other structures [56] which itself can aggravate kindling epileptogenesis [57].

Previous studies showed differential effects of CORT on early versus later stages of kindling [7], [58]. Our measurements of CORT early in kindling were timed to occur when the rats were having non-convulsive seizures (Class I–III), following the first 12 and 24 stimulations. This also minimised a potential confounding effect on stress responses of habituation due to handling. Our last CORT measurement, following a seizure (Class V) elicited two weeks after rats attained the ‘fully kindled’ state, showed that the enhanced HPA response following seizures was sustained. The early stage of kindling is a period of increasing cellular excitability [59], [60]. Epileptogenesis does not cease after clinical emergence of seizures [61], thus HPA hyper-reactivity, with enhanced seizure-induced CORT release, may be relevant to disease progression in established epilepsy.

### Exposure to Early Life Stress Is Associated with Decreased Hippocampal CA3c Pyramidal Cell Numbers Post-Kindling

While neuronal loss in a number of hippocampal and extra-hippocampal brain regions could potentially be relevant to the enhanced vulnerability to amygdala kindling of rats exposed to early life MS, CA3c was selected to be specifically examined in this study because it is thought to have a pivotal role in limbic epileptogenesis [4] and because a prior study employing electrical kindling and rigorous cell counting technique demonstrated cell loss in this region, amongst others [45]. Furthermore, the pyramidal neurons of CA3 have been shown repeatedly to be affected by stressors of various kinds, the effects including dendritic remodelling [2] and cell loss [62], although the later has been questioned in subsequent work [63], [64], [65].

In this study we found that rats exposed to early life MS had reduced pyramidal cell counts in hippocampal CA3c subregion compared with EH exposed rats, and that in female rats this was more marked in the kindled than sham kindled animals. Neuronal loss, even of excitatory neurons, may contribute to epileptogenic circuit re-organisation [5]. MS is known to affect hippocampal structure, particularly the DG [66], however there are no published data on the effects of MS alone on pyramidal cell numbers in adult life.

We speculate that the reduced cell numbers found in female rats exposed to MS are due to an early neurodevelopment effect of the MS combined with increased vulnerability to cell loss stemming from kindling. Given that there is no neurogenesis of hippocampal pyramidal cells in adulthood, the reduced cell numbers we observed cannot result from reduced cell production and so must result from cell death. If this is so, we propose two main candidate mechanisms to explain an increased vulnerability to kindling-induced cell death in MS-exposed rats. First, corticotropin releasing hormone (CRH) can injure CA3 hippocampal neurons [67], is upregulated by MS [29], [68] and, when experimentally elevated in infancy, reduces CA3c cell numbers in adulthood [69]. Second, the enhanced CORT levels post-seizure that we have shown here to occur in MS-exposed rats may aggravate glutamate-mediated excitotoxicity [54], [55].

Regarding cell numbers, there remain important questions to be resolved by future research. For example, we chose CA3c for initial study, for the reasons stated above, but clearly other structures are implicated in MTLE and show cell loss. These include CA1, dentate gyrus, dentate hilus and even extra-temporal structures. These too may be affected by MS stress and thus should be studied using quantitative stereological methods. In addition, elevated CORT from infancy has been shown to reduce cell numbers in CA3 at P30 and P180 [70], but this has been examined only in males, not females.

### Exposure to Early Life Stress Is Associated with Enhanced Kindling-Induced Neurogenesis

Amygdala kindling increases neurogenesis in the DG [71], [72], as do other animal models of epileptogenesis [73]. In contrast, MS typically reduces neurogenic responses [74]. This is the first study to examine the interaction between MS and kindling epileptogenesis on neurogenesis. We found that in female but not male rats MS was associated with augmented kindling-associated neurogenesis. Given that MS rats required fewer electrical stimulations to reach the fully-kindled state, one might expect less neurogenesis in this group; however, our data for female rats shows the opposite. No effect of MS on neurogenesis was seen in sham-kindled rats of either sex.

The greater neurogenesis in female MS kindled rats occurred despite markedly elevated CORT which typically suppresses neurogenesis [75], [76], [77], [78], and reduces survival and differentiation of newly-formed cells [79], [80]. Adrenalectomy stimulates proliferation [81], [82] and GR antagonists normalise neurogenesis in the presence of CORT [78]. Suppression of neurogenesis was related to elevated CORT levels induced by seizures in one study [83] but not another [76].

Mirescu et al. (Mirescu et al., 2004) found that MS-exposed rats had diminished neurogenesis in adulthood despite normal basal CORT levels, and also demonstrated that an acute, CORT-elevating stressor failed to suppress neurogenesis further. They did not find lower BrdU+ve cell counts in MS rats 3 weeks after a single BrdU administration – similar to our result using a 7-day BrdU protocol - but did find diminished counts in MS rats at 2 hours and 1 week after injection. These apparently conflicting findings suggest kindling-induced neurogenesis is modulated by CORT via a different mechanism than basal neurogenesis.

### Possible Interrelationship of Findings and ‘Two-Hit’ Models of Mesial Temporal Lobe Epilepsy

The currently held view of causation of MTLE is of a multistage process, i.e. in susceptible individuals, brain insults early in life trigger a cascade of neurobiological processes that ultimately – often after years or decades - result in an epileptic state marked by spontaneous recurrent limbic seizures [4], [84], a framework sometimes termed the ‘two-hit’ model. Thus, undoubtedly, multiple causal pathways are involved linking early stress to later epilepsy [17], [85], but we suggest the present findings are consistent with a large body of literature about pathogenic effects of sustained glucocorticoid release on limbic structures [6], [19], [21], [86]. Based on this literature, we speculate that early life stress ‘programming’ of HPA axis function produces hyper-reactivity, leading to exaggerated CORT release during kindling seizures. This hyper-reactivity in turn leads to greater kindling-associated augmentation of neuronal excitability, as well as to altered neuroplasticity (neurogenesis) and increased cell death, presumably due to enhanced cellular calcium influx. Interestingly, in the status epilepticus rat model of limbic epileptogenesis, another important animal model of human MTLE, heightened interictal CORT levels and responses (even without early life stress) were recently reported [87].

However, early life stress is known experimentally to have both short-term and long-term neurodevelopmental effects on limbic structures [26], [69], [70], [88], [89]. Thus life stress may act as a ‘hit’ at several stages in the ‘multi-hit’ process that is thought to give rise to MTLE [4], [5], [84].

### Sex Differences

Sex differences were not a primary focus of our research, but the sex differences we found here and in our previous study [15] merit comment. Univariate analysis by sex did not reveal statistically significant differences for any variable except seizure-associated CORT release, which was higher in females. However, only in female rats was maternal separation significantly associated with enhanced kindling-associated neurogenesis and with CORT elevation. In our previous study a significant effect of early life maternal separation stress to enhance the vulnerability to amygdala kindling epileptogenesis later in life was seen only in female rats [15], although in the current study this effect was seen in both sexes.

In seeking to understand these sex differences, several points are relevant. First, there is evidence that temporal lobe epilepsy is more prevalent in women [90], and that the clinical manifestations and neuroanatomical alterations (as seen on functional and structural imaging) are different. For example, in males, seizure spread is wider, secondary generalisation more common and the volume of hypometabolism more extensive [91], [92], [93]. Second, in animal studies, hormonal and behavioural stress responses are often greater or different in females [94] and cognitive effects dimorphic [95]. Furthermore, the effects on brain of stressors or stress mediators (such as CORT or CRH) are often sexually dimorphic [94]. In part, these differences may be dues to sex hormones, notably oestrogens [96] (also see discussion in [15]), which may protect against stress-induced effects on hippocampal morphology, including dentate neurogenesis [97], [98]. Thus oestrogens may protect against the neurogenesis-suppressing effect of the CORT release induced by kindling. However, the interaction between HPA function and gonadal steroid function is bi-directional: elevated CORT may suppress oestrogen levels acutely. Recently, sex differences in effects of MS on neurogenesis were reported using a different separation protocol and shorter follow-up [98]: females in all experimental groups had *lower* levels of neurogenesis than males and smaller granule cell layer volume. These conflicting results justify further research into these complex and important processes.

Stage of ovarian cycle is known to affect excitability, but cannot account for our findings as all critical procedures (notably kindling, CORT measurements and euthanasia to obtain brain) occurred in random relation to it. Thus stage of the ovarian cycle would have served as a source of variation tending to obscure true effects.

### Limitations

Some limitation of the study should be pointed out. First is the associative nature of the data; however, our findings are very clear and point to directly testable hypotheses which are amenable to experiments designed to test causality. Secondly, we designed the study as a ‘proof of principle’ experiment exploring the effect of contrasting stress exposures in early life on the later vulnerability to limbic epileptogenesis. Thus we employed contrasting exposures – MS and EH – that had been shown repeatedly to have opposite neurobiological and behavioural effects. However, it should be noted that there is an extensive debate in the early life stress literature about appropriate control groups for different research questions; we discussed this extensively in our initial publication [15]. Thirdly, for cell counting we employed the neuronal marker NeuN: NeuN expression can diminish after insults that do not lead to neuronal death [99], [100]. Thus, we have been careful in our discussion only to suggest - but not conclude - that cell death explains our findings. Having made our observation, an important next step is careful and systematic analysis of the basis of the reduced cell counts, including carefully timed use of cell death markers.

Strengths of the study are its use of the most commonly studied rat model of mesial temporal lobe epilepsy, the amygdala kindling model [101]; of a standard, well characterised form of early life stress (MS); and of rigorous cell counting technique. Although early life stress has been implicated in rodent models of limbic epilepsy, studies of intervening mechanisms remain rare [17].

Overall, our study provides novel observations suggesting an important role for CORT in the augmentation of limbic epileptogenesis by early life stress and points to mechanisms by which it may be acting, namely by enhancing hippocampal pyramidal cell loss and dentate granule cell neurogenesis. The data are associative in nature but provide strong guidance for future causal testing in animal models. Given the high prevalence of chronic stress states in humans, and the high prevalence of comorbid depression (itself a stressful state, often with elevated cortisol) [102], treatment with GR antagonists or corticosteroid synthesis inhibitors may be of utility in MTLE patients.
